# Consumers’ Behavioral Willingness to Use Green Financial Products: An Empirical Study within a Theoretical Framework

**DOI:** 10.3390/bs14080634

**Published:** 2024-07-24

**Authors:** Xiangwei Xie, Chunxi Gong, Zhenqing Su, Yufei Nie, Woohyoung Kim

**Affiliations:** 1 School of Business, Southwest University, Chongqing 402460, China; xiexiangwei8@swu.edu.cn; 2 The Graduate School of Technology Management, Kyunghee University, Global Campus, Yongin-si 17104, Republic of Korea; gcx2021725213@khu.ac.kr (C.G.); yufei@khu.ac.kr (Y.N.); 3 The Graduate School of Global Business, Kyonggi University, Suwon-si 16227, Republic of Korea; suzhenqing@kyonggi.ac.kr; 4 The Graduate School of Technology and Innovation Management, Hanyang University, Seoul 04763, Republic of Korea

**Keywords:** Ant Forest, green financial products, customer-perceived value, TAM, consumer behaviors

## Abstract

In an era marked by the expansion of the Internet economy and the intensification of environmental concerns, the convergence of digital finance and green finance has emerged as a significant global trend. China’s Alipay Ant Forest, an innovative green financial product, has successfully quantified carbon emission reductions resulting from users’ green consumption patterns, establishing the first carbon account-based green financial product and pioneering an innovative “green finance plus gamification” model. However, the academic literature has not fully explained the underlying mechanisms that drive consumer engagement with such green financial products. This study, motivated by the academic question of what factors influence consumers’ willingness to use green financial products, employs Ant Forest as a case study and develops a novel structural equation model based on self-determination theory, customer-perceived value, and the technology acceptance model. The model incorporates user type as a control variable and considers autonomy, gamification, and bonuses as key independent variables, with customer-perceived value serving as a mediating variable. Data collection involved 606 participants, enabling a comprehensive analysis of the factors influencing users’ willingness to engage with green financial products. The findings support the proposed hypothesis, identifying several significant predictors of users’ willingness to use green financial products, with the exception of age. This study advances the theoretical understanding of consumer behavior towards green financial products by integrating self-determination theory, customer-perceived value, and the technology acceptance model, while also offering practical insights for marketing strategies. It explores the interface between digital finance, environmental sustainability, and consumer behavior, highlighting opportunities for financial institutions to leverage Internet applications to promote green financial services and enhance their marketing approaches to influence consumer adoption.

## 1. Introduction

In recent years, with the rapid advancement of technology and the increasing consumption of resources, environmental damage has become a significant concern [1]. Governments worldwide have heightened their focus on environmental protection, climate change, and sustainable development, setting relevant climate goals. The Chinese government, for instance, pledged at the United Nations General Assembly in September 2020 to peak carbon emissions by 2030 and achieve carbon neutrality by 2060. However, despite government commitments and policies achieving some results, individuals are still not held directly accountable for environmental changes [2].

Notably, research indicates a direct link between environmental changes and human behavior [3,4]. Therefore, to improve the environment, it is necessary first to change individual human behavior. Furthermore, in the context of the Internet economy and society, an increasing number of countries are choosing to address social issues through financial means [5]. Meanwhile, online social media subtly influences people’s lifestyles, perceptions, and behaviors [6,7]. Consequently, with the growing severity of environmental issues and the promotion of sustainable development concepts, Internet-based green financial products have gradually emerged and are increasingly gaining the attention of policymakers and researchers [8]. Specifically, green financial products support individual consumer behaviors in environmental protection, resource conservation, and pollution control by allocating financial resources, thereby tightening the relationship between green finance and individuals and offering new solutions to the problem of continuous environmental consumption [9].

Among green financial products, Ant Forest is a representative online green financial product that integrates public welfare, gaming, and social interaction, making it the most widely used online green environmental financial platform globally [10,11]. By gathering individual efforts, Ant Forest contributes to the development of green finance and provides new answers to the ongoing issue of environmental consumption [12].

Despite its significant success, the key to the sustainable development of Ant Forest lies in continuous consumer use [13]. Therefore, understanding Ant Forest users’ behavior and willingness to continue using Ant Forest is crucial. The main aim of this paper is to explore the factors influencing consumers’ behavior and willingness to use green financial products based on Ant Forest. Many online applications aim to raise public awareness of environmental issues, but few are specifically designed for this purpose [14]. Thus, in addressing these research questions we apply self-determination theory and establish a structural equation model with autonomy, gamification, and rewards as independent variables and customer-perceived value as a mediating factor to explore the influence on consumers’ willingness to use green financial products. This model was empirically tested through a survey of Ant Forest users.

This study theoretically enhances our comprehension of consumer behavior towards green financial products by incorporating self-determination theory, customer-perceived value, and the technology acceptance model. It offers a more refined understanding of the underlying factors influencing consumers’ propensity to adopt green financial products, thereby enriching the existing theoretical research on the subject. Moreover, the exploration of the integration of green finance with Internet applications provides novel insights into the potential convergence of these two domains, filling a significant gap in current research. This integration has the potential to revolutionize the green financial market by leveraging the power of digital technologies, thereby offering new opportunities for financial institutions, policymakers, and managers. The findings of this study can also inform marketing strategies for green financial products, helping financial institutions and managers enhance their marketing efforts. By understanding the factors influencing consumers’ willingness to use green financial products, they can tailor their marketing approaches to effectively target potential customers, thereby increasing the uptake of green financial services.

## 2. Literature Review and Hypothesis Development

### 2.1. Green Financial Products and Ant Forest

In recent years, since [15] first proposed the concept of green finance, it has garnered widespread interest among scholars [16,17,18,19,20]. Green finance is considered a key factor in promoting environmental progress and achieving sustainable development [21,22,23,24]. As an innovative model that organically combines traditional financial instruments with environmental protection policies, green finance differs from the projects served by traditional financing [25], effectively adjusting the social industrial structure and providing strong financial support for the development of green industries and technological advancement [26]. However, the academic community has yet to reach a consensus on green finance [27].

Early research primarily focused on the relationship between green finance and the economy itself [16,22]. Subsequently, with the expansion of the scale of green finance investment, the main forms of green finance have transformed into green credit, green insurance, and green banking [23]. Some scholars focus on the impact of green finance on resource utilization. Among them, some scholars have found that green finance effectively supports clean energy, promotes efficient resource utilization, and encourages the application of renewable energy [18,19]. Based on this, [24] found a strong bidirectional causal relationship between green finance and clean energy throughout the analysis period, indicating that clean energy is also driven and influenced by green finance investments.

Meanwhile, with the combination of green finance theory and Internet technology, many innovative green finance practice products have emerged. Among the many green financial products, Ant Forest adopts an innovative “public welfare + gamification” model, applying it to the product and balancing public welfare value with market economic value [25]. It quantifies the carbon emission reductions from users’ green consumption behaviors into digital measurements, forming users’ exclusive carbon accounts, encouraging everyone to contribute to energy conservation and emission reductions through their carbon account platforms [26], and converting them into carbon assets through financial means, effectively promoting the development of personal carbon finance products in green finance [27]. Therefore, many scholars have studied Ant Forest as an innovative practice for successful green financial products [28,29].

**Figure 1:**
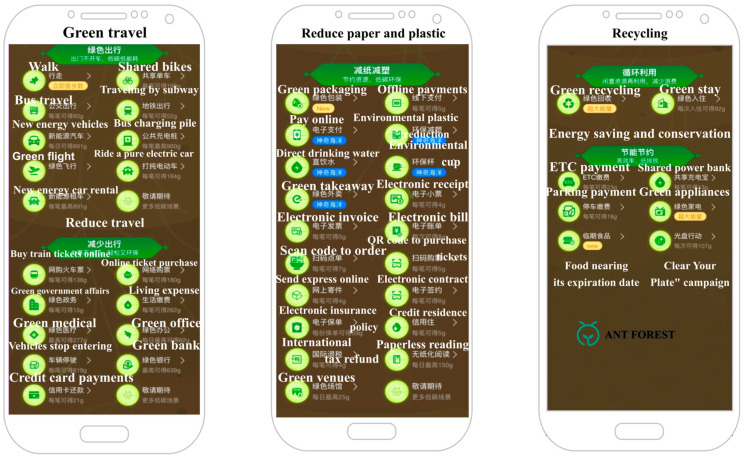
Ways of gaining energy demonstrated in Ant Forest. Source: screenshot from Ant Forest.

Specifically, in 2016, Ant Group utilized the Alipay platform in China to create a personal carbon account for customers called “Ant Forest”, motivating users to take low-carbon environmental actions. Ant Forest converts users’ green consumption and low-carbon activities on Alipay into “green energy”, which feeds back into the online game through users’ offline low-carbon behaviors [29]. The primary goal for Ant Forest users is to accumulate a certain level of “green energy” to exchange for various types of virtual trees. Figure 1 illustrates the various methods of obtaining energy in Ant Forest. After planting virtual trees, the Ant Group plants a real tree in an ecological area. As an incentive for continuously reducing their carbon footprint, users receive a green certificate and can view images of the trees via satellite in real time [12].

**Figure 2:**
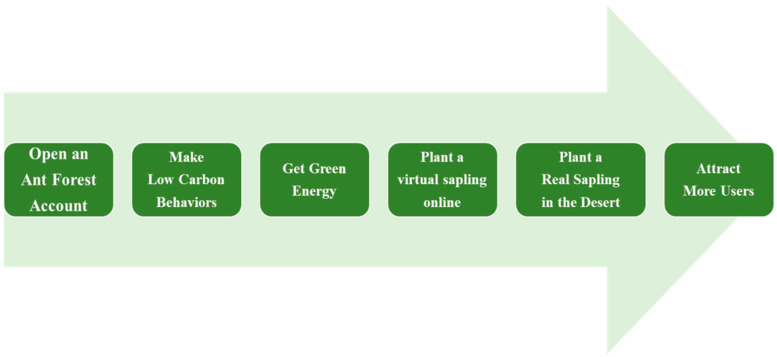
Ant Forest green results flow chart.

Moreover, Ant Forest has social attributes, allowing users to “steal” green energy from friends, help water friends’ trees, and even plant trees together [27,30]. Accumulating a certain amount of energy can be exchanged for different types of trees. The relationship between the amount of green energy in the carbon account and the exchange of various tree seedlings is shown in Table 1. Through this gamification approach, users can engage in low-carbon behaviors [14] and increase social interaction [28]. Ant Forest uses the virtual seedlings planted by users to achieve offline interaction, create social impact, and attract new users (as shown in Figure 2), thereby achieving a closed loop. This is an innovative development.

In 2017, Ant Group and the United Nations Environment Programme launched the Green Digital Finance Alliance in Davos [5]. In 2019, Alipay Ant Forest won the United Nations’ highest environmental honor, the Champions of the Earth Award [10]. According to the “Ant Group 2021 Sustainability Report”, as of 2021 there are now more than 600 million Ant Forest users, planting a cumulative total of 326 million trees and reducing carbon emissions by over 12 million metric tons.

Despite the significant success of Ant Forest, the key to its sustainable development lies in continuous consumer use [14]. However, existing research on Ant Forest mainly focuses on consumer attitudes and behavior, psychological factors and willingness, and exploring the role and function of Ant Forest as a green platform. Few studies have systematically examined consumer behavior and willingness to use green financial products within the framework of Ant Forest. Furthermore, in the realm of green financial products, greenwashing risks should not be overlooked, as they significantly reduce consumers’ willingness to use green financial products [31].

### 2.2. Theoretical Framework

**Figure 3:**
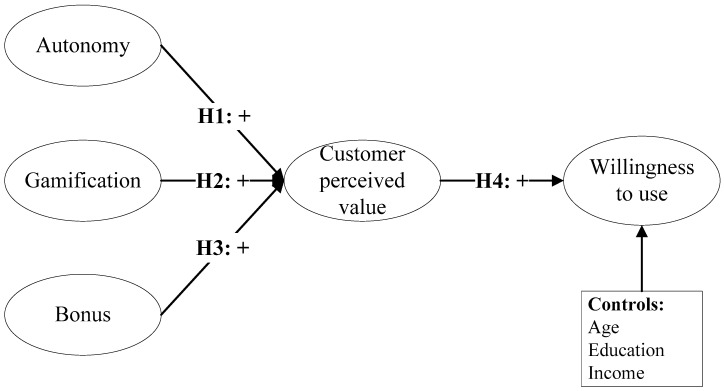
The theoretical framework.

This research model is based on self-determination theory, perceived value theory, and technology acceptance model theory to explain the influence of consumers on their intention to use green financial products. Figure 3 shows the theoretical model and hypothetical relationship of this study. With autonomy, gamification, and rewards as independent variables, customer-perceived value plays a mediating role in influencing customers’ willingness to use green financial products. Additionally, take the user type as the control variable. The theoretical basis for this model is discussed in the next section.

### 2.3. Self-Determination Theory

At present, self-determination theory is frequently employed by academics to explain why individuals engage in gamification marketing activities. According to self-determination theory, individuals engage in activities for the following three primary reasons: intrinsic motivation, extrinsic motivation, and internalized motivation [32]. Self-determination theory posits that intrinsic motivation has to do with things that arise out of intrinsic interest in themselves, rather than extrinsic, separable outcomes. Gamification incorporates elements of games to fulfill individuals’ psychological needs and stimulate internal motivation [33,34]. The self-need theory proposes that human beings generally have three basic psychological needs: autonomous needs, related needs, and competent needs. The satisfaction of these three psychological needs is the basis for the healthy growth and self-development of individuals [33]. A game itself can stimulate a player’s sense of autonomy, competence, and ability to relate emotions, such that a player actively and voluntarily participates and invests in it [35,36].

#### 2.3.1. Autonomy

According to self-determination theory, autonomy is a user’s psychological need to act freely with the feelings of a protagonist [37]. Autonomy serves as the root of an individual’s behavior, and the need for autonomy is internally driven rather than externally imposed [38]. In general, autonomy is achieved by users, as individuals need to feel a sense of ownership over their actions and feel psychologically free. The need for autonomy was the focus of early self-determination theory research, as it was shown to help explain the adverse effects of extrinsic motivation on the emergence and persistence of intrinsic motivation [39]. Intrinsic needs for autonomy are fulfilled when individuals recognize that they are the origin of their choices and decisions and that their actions are consistent with their whole sense of self [38].

According to Weiner’s attribution theory, the more an individual is involved in the decision-making process for an event, the more the outcome is seen as reflecting an individual’s own reasons [40]. Additionally, there are some studies in the field of games that believe a game itself can stimulate players’ sense of autonomy, such that they can participate in it actively and voluntarily [35]. Therefore, the following hypothesis is proposed:

**H1.**  *The degree of user autonomy positively affects the perceived value of consumers using Ant Forest.*

#### 2.3.2. Gamification

The term gamification was originally coined by Pelling [41]. By incorporating game elements into the user interface of commercial electronic equipment (e.g., ATMs), he implemented a gamified user interface design to increase the variety and efficiency of electronic transactions [42]. 

The most widely accepted definition of gamification was proposed in 2011 as the integration of game design elements into non-gaming environments [43]. Studies increasingly focus on applying gamification to software platforms, defining it as the incorporation of game elements into non-gaming software to enhance user experience and engagement [44]. Gamification also covers marketing concepts such as brand awareness, service, motivation, ownership, or purchase intent [45,46]. Gamification can enhance users’ willingness to engage [47,48] and increase their satisfaction with as well as loyalty to retailers [49]. Therefore, the following hypothesis is put forward:

**H2.**  *Gamification factors have a positive impact on users’ perceived value.*

#### 2.3.3. Bonus

A bonus is frequently defined as money, tangible prizes, or symbolic items of real achievement, such as trophies [50]. We can divide bonuses into tangible and intangible categories based on their manifestations. For instance, gamification frequently employs bonuses such as point prizes or game currency for rewards, rather than using actual cash payments. We can classify the manifestations of bonuses into two categories: tangible and intangible [51]. In Ant Forest, users receive bonuses in the form of “green energy” as a reward for their environmental protection behavior. Users also receive an environmental certificate as a bonus.

In many online games, as players progress, they are rewarded with higher virtual rewards, such as game points or virtual currency. These rewards help players play the game better and can increase perceived value and customer loyalty [52]. The extra rewards obtained in the game may cause users to feel that the game is worth the money [53]. According to [54], people like to learn but tend to be lazy. In games, this means that players tend to receive bonuses. The authors of [55] argued that reward systems should keep players excited during the game. Many researchers, such as those of [56,57], believe that the pleasure of anticipation is an important aspect of positive experience. In the context of video games, players experience pleasure in anticipation of rewards [58]. The positive impact of reward acquisition (or motivation gratification) on consumers’ psychological experience of sporting events has been well documented [59,60]. Moreover, research has indicated that customers will be more inclined to utilize a website on a regular basis due to the incentive system incorporated into a game [61]. Therefore, we hypothesize the following:

**H3.**  *The bonus positively affects consumers’ perceived value.*

### 2.4. Customer-Perceived Value and Technology Acceptance Model

The author introduced the concept of customer-perceived value (CPV), defining it as the value that customers assign to a product or service based on the benefits perceived and the cost incurred to obtain it, culminating in an overall service utility assessment [62]. Subsequent research, including contributions from [63,64,65], has identified both the sources of CPV and its constituent elements. The authors of [63] expanded on this by emphasizing the role of context, alongside objective factors like quality as well as price and subjective customer factors, in shaping CPV. Perceived value is recognized as a critical driver of users’ continued engagement with mobile technology [60] and influences their intentions to continue using services [66].

The authors of [67] developed the technology acceptance model (TAM) based on the theory of reasoned action, aiming to explain how users adopt new technologies. According to [68,69], a user’s intention to engage with technology is largely determined by its perceived usefulness and ease of use. The TAM has been extended from traditional information systems to the digital realm and has been widely applied in studies, including those on Internet gaming behaviors. For instance, [70] used the TAM to examine the influence of social impact and immersive experiences on gaming, while [71] integrated the TAM with the theory of planned behavior to investigate online gaming persistence.

In the realm of green environmental protection, research has explored users’ perceived risks in online banking and the drivers of continued gaming engagement as well as in-app purchases [70,72,73]. The authors of [66] have provided a model suggesting that all forms of perceived value positively impact continuation intentions, with game quality and monetary value encouraging in-game purchases. Based on these findings, the following hypothesis is proposed:

**H4.**  *Customer-perceived value positively affects willingness to use.*

## 3. Research Methodology

### 3.1. Survey Design

The proposed research hypothesis was examined in this paper by administering a questionnaire survey to gather data. At the beginning of the questionnaire, there is an introduction that is about the research background, research purpose, and confidentiality guarantee to the interviewees.

The questionnaire design and survey execution are based on the research framework constructed above and the hypotheses proposed in this paper. To meet the present situation, according to the actual research object, Ant Forest, the measurements for every variable have been edited from the present literature. Given that variables are not readily observable or measurable, this paper has designed multiple test items for each variable. Additionally, to collect data more accurately and productively, the scoring method of the scale adopts a Likert seven-point scoring method, from strongly disagree to strongly agree, and the score is given from 1 to 7 according to the answer. The specific content of the scale is shown in Table 2.

The questionnaire is structured into two main sections. The initial section gathers demographic data from participants, encompassing gender, age, educational level, monthly income, occupation, and the frequency with which they use Ant Forest. Table 2 presents the specific items respondents are tasked with evaluating in the second section. To ensure the accuracy of responses, an attention check question is included. Prior to commencing the second section, an instruction is provided for respondents to select the number “5”, ensuring that only data meeting this criterion are considered valid. Recognizing that Ant Forest is a Chinese application with a predominantly Chinese user base, and to facilitate better comprehension among respondents, the questionnaire is translated into both English and Chinese. 

### 3.2. Data Collection

The survey period for this questionnaire spanned from October 2022 to November 2022. Due to geographical reasons, all questionnaires for this survey were distributed online. The Wenjuanxing questionnaire platform, one of the most popular online platforms in China, was employed for the design and input of the questionnaire content, generation of questionnaire links, and distribution on major social network platforms. The aforementioned platforms included WeChat Moments, WeChat group chats, Ant Forest users’ QQ group chats, and the Alipay chat room. The survey was conducted in Shanghai, China, and a total of 606 valid questionnaires were collected (586 people who have used “Ant Forest” and 20 people who have not used “Ant Forest”). The data were subjected to factor analysis and structural equation modeling in order to investigate them.

As per the research inquiries and subjects of investigation in this paper, the questionnaire survey is subject to certain stringent limitations. To ensure the validity of the questionnaire and ensure that the respondents who fill it out are all users of Ant Forest, the questionnaire has set the first question as a particular question: “Have you ever used Ant Forest?” If a participant chooses “no”, the survey will end directly. Meanwhile, the questionnaires were distributed to ensure a balance between males and females as much as possible. In the end, after sorting out the questionnaire data, removing invalid questionnaires that failed to pass the screening questions, questionnaires that took less than 60 s to have all the questions answered, and questionnaires that were I-shaped (referring to respondents’ tendency to provide answers aimed at shaping how they are perceived by others rather than reflecting their true thoughts or behaviors) and Z-shaped (referring to respondents’ inclination to select agree or disagree options in a predetermined pattern, often without genuine reflection on the specific content of the questions; this behavior occurs without detailed analysis or consideration of the question’s context), a total of 606 questionnaires were collected in this survey. The questionnaire was validated and the data were used for analysis.

### 3.3. Bias Tests

The dataset used in this study, collected via a self-report method, may introduce two bias issues: nonresponse and common method bias. To assess the potential nonresponse bias, we compared the responses of early and late respondents by using a one-sample *t*-test. The insignificant results indicate that nonresponse bias is unlikely to be a problem in the present study. Next, we used Harman’s one-factor test to determine the likelihood of common method bias, despite the careful design of our questionnaire, such as adding screening questions and creating multiple vignettes and random items [74]. The results of the total variance of the first single factor were below the suggested threshold value of 50% (36.86%), suggesting that our sample did not suffer from common method bias. Finally, we further checked the multicollinearity problem by testing the variance inflation factor (VIF) of the variables, and the results showed that the VIF values of our sample were below the cut-off value of 3.3 (2.085–2.772). Therefore, the sample used in the present research was free from multicollinearity problems.

## 4. Results

### 4.1. Demographic Characteristics

Table 3 displays the demographic features of the survey sample. In terms of age distribution, the plurality of respondents fell between the age range of 20 and 29 years, comprising 40.1% of the total. Additionally, 73.4% of the respondents were below the age of 40. This aligns closely with the attributes typically exhibited by consumers of third-party payment services in China. Based on the most recent statistics obtained from the Internet, the most recent statistical data from the Internet indicate that the age distribution of China’s mobile payment users remained largely unchanged in 2022. Out of the total users, 70% were in the age range of 18–40, while the remaining 30% were above the age of 40 [75]. In terms of educational background, more than half had a bachelor’s degree, making up 65% of the sample. Relatively speaking, this group has a higher level of education, more active thinking, finds it easier to accept new things, and has higher cultural literacy [76]. These traits are inferred from the educational attainment of the respondents rather than directly assessed in this study.

Therefore, taking them as a sample can better reflect the characteristics of network user interaction and participation in public welfare, which is consistent with the purpose of this research. Regarding the use of Ant Forest, the respondents in this survey have a high degree of participation in Ant Forest. Of the respondents, 15.3% used Ant Forest five or more times a week, 36.1% used Ant Forest three–four times a week, and 45.2% used it once or twice a week. These statistics show that the frequency of respondents’ use of “Ant Forest” is increasing.

Furthermore, a total of 606 individuals participated in the survey, and 20 respondents indicated that they had no previous experience with “Ant Forest”. As a result, these 20 survey samples were excluded from the subsequent process of analyzing the data.

### 4.2. Measurement Model Analysis

Confirmatory factor analysis was employed to examine the constructs’ reliability, validity, and overall fitness, and the findings are presented in Table 4 and Table 5. The results in Table 4 suggest a good model fit based on the ratio of the chi-square value to the degree of freedom (χ^2^/df), incremental fit index (IFI), comparative fit index (CFI), Tucker–Lewis fit index (TLI), and root mean square error of approximation (RMSEA). Model fit indices: χ^2^/df = 1.533, CMIN = 245.266, df = 160, IFI = 0.988, CFI = 0.987, TLI = 0.985, and RMSEA = 0.030.

Moreover, the reliability of our model is ensured as the standardized factor loadings (0.773–0.845), Cronbach’s alpha values (0.858–0.895), and composite reliability (CR) (0.858–0.895) of the constructs are all above 0.70. In addition, the results in Table 4 also support the convergent validity of our measurement model because all average variance extracted (AVE) values are found to be greater than the recommended criteria of 0.50. We check for the discriminant validity by comparing AVE values and their squared correlations with other constructs. In Table 5, all AVE values are higher than the squared correlations. Therefore, discriminant validity is also achieved in this study.

### 4.3. Hypothesis Testing

**Figure 4:**
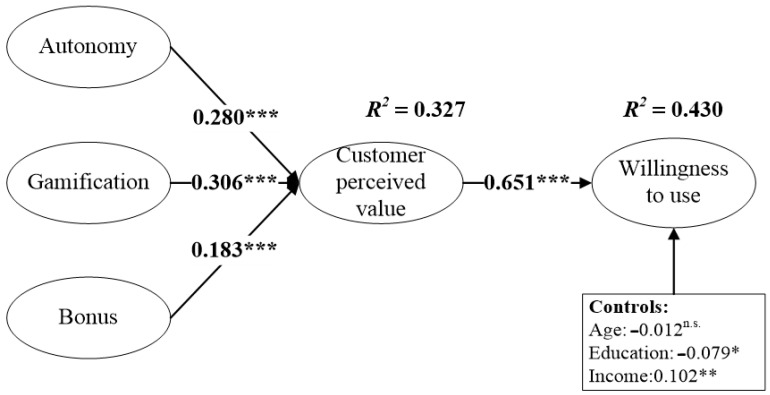
Hypothesis test results. Note: model fit indices: c2/df = 2.689, CMIN = 438.303, df = 163, IFI = 0.960, CFI = 0.960, TLI = 0.953, and RMSEA = 0.053. n.s. *p* > 0.05, * *p* < 0.05, ** *p* < 0.01, and *** *p* < 0.001.

We examined the proposed hypothesis using structural equation modeling analysis; the results are in Figure 4. Note that we considered the model’s control variables, including age, education, and income, to control external effects. As expected, numerous fit indices, χ^2^/df = 2.689, CMIN = 438.303, df = 163, IFI = 0.960, CFI = 0.960, TLI = 0.953, and RMSEA = 0.053, support the structural model’s overall fit. In addition, the standardized path coefficients for all structural paths were statistically significant at a 99% confidence level, whereas the impact of age (b = −0.012 n.s.) on WTU was insignificant. The results show that our model explains 32.7% of the variance in customer-perceived value (CPV) and 43% of the variance in willingness to use (WTU), as indicated by the R^2^ values.

According to Figure 4, the impact of AUT on CPV (b = 0.280 ***) was significantly positive, providing strong support for H1: the degree of user autonomy positively affects consumers’ perceived value. In addition, the results showed a significant positive relationship between GAM and CPV (b = 0.306 ***), which validates H2: gamification attributes positively affect consumers’ perceived value. We examined H3 by estimating the influence of BON on CPV, and the results suggest that the path coefficient was significant and positive (b = 0.183 ***). Therefore, these findings support H3: the reward mechanism positively affects consumers’ perceived value. To investigate H4, we tested the impact of CPV on WTU, and the result suggests that the standardized coefficient was significantly positive (b = 0.651 ***), suggesting support for H4: customer-perceived value positively affects willingness to use.

We further explored the indirect effect between variables using mediation analysis (Table 6). We employed the bootstrapping method using 5000 replications with bias-corrected confidence intervals. Our results do not yield any negative values regarding the 95% confidence intervals, generating support for indirect effects. Specifically, we found that CPV fully mediated the impact of AUT (bind = 0.182, boot SE = 0.038 ***), GAM (bind = 0.199, boot SE = 0.037 ***), and BON (bind = 0.119, boot SE = 0.036 **) on WTU.

## 5. Discussion

We utilized the “Ant Forest” app as a case study to delve into the behavioral drivers behind consumers’ decisions to embrace green financial products, employing a structural equation model informed by self-determination theory. In this paper, a questionnaire survey was conducted in Shanghai, China, and 606 valid questionnaires were collected. Validated factor analysis and structural equation modeling were used to investigate the data. All three independent variables—autonomy, gamification, and bonus—contribute to users’ willingness to use green financial products. Specifically, when users feel a great deal of autonomy in using green financial products and receive some bonuses through gamification, their perceived value is enhanced, which increases their willingness to use them.

Furthermore, gamification has the most significant impact on the willingness to use green financial products. To a certain extent, some gamification attributes can be added to green financial products to increase users’ autonomy in using these products, and some reward mechanisms are constantly added. The products can effectively motivate users to use green financial products. Overall, this study provides valuable insights and makes theoretical and managerial contributions.

### 5.1. Theoretical Contributions

This paper contributes to the theory of green financial products by expanding the scope of research from specific domains to green financial applications in the digital domain [77]. Taking Alipay Ant Forest as an example, we developed a structural equation model that includes self-determination theory, customer-perceived value, and a technology acceptance model. The model elucidates consumers’ behavioral willingness to adopt green financial products. It also explores the relationship between these products and consumer behavior, thus enriching the literature on the behavior of consumers using green financial products on the Internet. Second, although previous studies on consumers’ green product behavior have been extensive, they have mainly focused on analyzing the purchasing behavior of green products, such as organic food, green consumer goods, energy-saving home appliances, green hotels, etc. [7,23,78]. Researchers should pay more attention to studies in the fields of consumer willingness and green financial products, especially consumer usage behavior. Therefore, similarly to the studies of [7,78], we take consumer usage intention as an entry point to expand the framework of consumer behavior and green financial products in a more in-depth way and to enrich the research on consumer behavior and green financial products. Third, we establish a broader literature link between consumer usage intention and green financial product research and integrate theoretical research into green financial product research. Specifically, based on [28]’s understanding of Ant Forest from the perspective of behavioral reasoning theory and other understandings of users’ behavior towards Ant Forest applications, we start with the impact of features such as autonomy and gamification on consumers’ perceived value and explore the impact of customers’ perceived value on consumers’ willingness to use Ant Forest, which has rarely been mentioned in previous research on Ant Forest. Therefore, by examining consumer behavioral intentions in the context of green financial products, this study not only fills a gap in the existing literature but also provides empirical evidence and insights to promote the adoption of green financial behaviors.

### 5.2. Managerial Contribution

From a managerial point of view, our study may provide some favorable assistance to relevant managers for developing and marketing green financial products on the Internet. This study may increase the attention paid by financial institutions and policymakers to the use of green financial services by consumers and provide some ideas for the future development of green financial services. First, the results suggest that green financial products can increase users’ willingness to use the products by enhancing consumers’ perceived value. Consumers with higher perceived value are more motivated to use green financial products, so regulatory agencies must pay attention to the importance of consumers’ perceived value. Secondly, among the factors that may increase perceived value, gamification significantly impacts willingness to use. Bonuses and autonomy significantly influence users’ willingness to engage with green financial products by enhancing perceived value, which, in turn, positively affects their likelihood of using such products. All three independent variables increase users’ willingness to use green financial products. Therefore, managers of green financial products can increase consumers’ willingness to use them by designing games with more liberalization and higher bonuses for completing green financial behaviors. Taking Ant Forest tree planting as an example, managers can provide more choices for users, who can match and decorate the saplings that they plant according to their preferences. To enhance the attractiveness of the bonus mechanism, consider implementing a system where users are rewarded with different bonuses based on the unique combinations of tree varieties that they plant. This approach would encourage users to explore and experiment with diverse planting combinations while also incentivizing them to engage more deeply with the platform. The proposal to offer varied bonuses for planting particular tree combinations in Ant Forest is designed to boost user enthusiasm and encourage ongoing participation. Third, this approach could serve as a valuable strategy for governments and environmental organizations looking for innovative methods to inspire public involvement in environmental efforts. With the Internet platform and Ant Forest’s rich game forms and bonuses, it can not only break the time and space limitations of offline environmental activities but also attract more people to use green financial products. At the same time, governments and environmental organizations can use Internet technology to adjust game formats to better spread green behaviors and low-carbon lifestyles, fundamentally change consumers’ habits and willingness to use green financial products, and promote the effective promotion of green financial products. By fostering active public participation in online environmental protection initiatives, this approach helps to embed the social behavioral norms of sustainable development and environmental stewardship deep within the hearts and minds of the populace.

### 5.3. Limitations and Future Research

The sample for this study is predominantly composed of individuals aged 20 to 40, a significant proportion of whom are students pursuing undergraduate or postgraduate degrees. On the other hand, Ant Forest is a small program embedded in Alipay and is used by many people. The participants’ age range might not be fully representative, so the selected sample may only partially reflect the user base of Ant Forest. Future research could consider a more diverse sample in terms of both education and age to enhance representativeness. The second limitation is the scope of the study. On the one hand, the data for this study were collected in Shanghai, China, and due to geographical and cultural constraints we were unable to consider geography comprehensively. On the other hand, the respondents of the empirical study are the users currently using Ant Forest, and they have no requirements on the depth or degree of use. However, some questions in the questionnaire require respondents to have a more in-depth understanding of Ant Forest to answer objectively. Therefore, the 606 questionnaires collected may not fully represent the usage intentions of Ant Forest’s 600 million users. The results of the study only represent the preferences of some participants. In addition, the context of this study is Ant Forest, and it remains to be investigated whether our findings can be applied to other similar behavioral applications of green financial products on the Internet. Finally, when developing green financial products, the risk of greenwashing can significantly reduce consumers’ willingness to use these products, thereby affecting their overall benefits. Therefore, the risk of greenwashing can be effectively mitigated by introducing independent third-party certification, standardized indicators, transparent disclosure, and technological innovation, ensuring the real benefits of green financial products.

## Figures and Tables

**Table 1:** The exchange relationship between the amount of green energy in the carbon account and different tree seedlings.

Green Energy	Tree Name (Latin Name and Chinese Name)
16,930 g	*Caragana korshinskii* (Kom.) and Ningtiao
17,900 g	*Haloxylon ammodendron* (C. A. Mey.) Bunge. and Suosuo
21,310 g	*Hedysarum mongolicum* (Turez.) and Yangchai
21,310 g	*Hedysarum scoparium* (Fisch. & C. A. Mey.) B.Fedtsch. and Huabang
38,570 g	*Armeniaca sibirica* (L.) Lam. and Shanxing
96,000 g	*Platycladus orientalis* (L.) Franco and Cebai
114,000 g	*Pinus tabulaeformis* (Carr.) and Yousong
146,210 g	*Pinus sylvestris* L. var. mongholica (Litv.) and Zhangzisong
198,000 g	*Picea asperata* (Mast.) and Yunshan

(Source: summarized from Ant Forest).

**Table 2:** Scale development.

Research Variable	Question (Strongly Disagree (1)/Strongly Agree (7))	References
Autonomy	(Aut_1) Ant Forest provides many ways and methods to complete the game goals, I can choose freely.	[27,29]
	(Aut_2) Ant Forest makes it possible for me to make personal decisions in its activities.	
	(Aut_3) Through the ant forest rankings, I can show my public welfare achievements.	
	(Aut_4) If possible, I will interact more with my friends in Ant Forest.	
Gamification	(Gam_1) I think Ant Forest’s player leaderboard design is interesting.	[13,14]
	(Gam_2) Through the ant forest rankings, I can show my public welfare achievements.	
	(Gam_3) I think the game of Ant Forest is very interesting.	
	(Gam_4) Compared with other green financial platforms, the gamification method of Ant Forest is more attractive to me.	
Bonus	(Bon_1) I try to get more medals, environmental certificates as my activity reward.	[28]
	(Bon_2) I am trying to get more green energy as my activity reward.	
	(Bon_3) I’m trying to have a higher leaderboard rank as a reward for my event.	
	(Bon_4) The Rewards of Ant Forest can motivate me to use it for a long time.	
CPV	(CPV_1) I think the concept that Alipay wants to convey is the core of the Ant Forest game.	[29,30]
	(CPV_2) I think Alipay’s image is related to the theme of Ant Forest.	
	(CPV_3) I can feel the concept that Alipay wants to convey from Ant Forest.	
	(CPV_4) Overall, I am satisfied with the design of Ant Forest.	
Willingness to use	(WTU_1) In the future, I would like to continue to participate in the public welfare activities of Ant Forest.	[21,28]
	(WTU_2) I think that by using Ant Forest increased my willingness to use Alipay.	
	(WTU_3) My plan is to keep playing Ant Forest and not choose other similar apps.	
	(WTU_4) I would like to introduce Ant Forest to my relatives and friends.	

**Table 3:** Respondent demographics.

Items	Category	Frequency	Percentage (%)
Age (years)	<20	84	13.9
	20–29	243	40.1
	30–40	202	33.3
	>40	77	12.7
Education	Middle school or below	12	2.0
	High school	175	28.9
	Bachelor	394	65.0
	Postgraduate or above	25	4.1
Monthly income (CNY)	<3000	20	3.3
	3001–5000	274	45.2
	5001–8000	219	36.1
	>8000	93	15.3
Usage experience	Yes	586	96.7
	No	20	3.3
Usage frequency (weekly)	0	20	3.3
	1–2	274	45.2
	3–4	219	36.1
	>5	93	15.3
Gender	Male	291	48.0
	Female	315	52.0

Note: N = 606.

**Table 4:** Discriminant validity.

	AUT	GAM	BON	CPV	WTU
AUT	0.826 ^a^				
GAM	0.278 ^b^	0.813			
BON	0.320	0.357	0.799		
CPV	0.378	0.413	0.337	0.785	
WTU	0.576	0.524	0.544	0.600	0.776

Note: ^a^ square root of AVE values is along the main diagonal; ^b^ below the main diagonal lists the correlations between constructs.

**Table 5:** Confirmatory factor analysis results.

Construct	Item	Mean	SD	λ	Alpha	AVE	CR
Autonomy (AUT)	AUT1	4.868	1.512	0.817	0.895	0.682	0.895
	AUT2	4.871	1.484	0.845			
	AUT3	4.883	1.483	0.821			
	AUT4	4.870	1.501	0.819			
Gamification (GAM)	GAM1	5.149	1.310	0.811	0.886	0.661	0.886
	GAM2	5.157	1.311	0.796			
	GAM3	5.150	1.314	0.821			
	GAM4	5.158	1.280	0.823			
Bonus (BON)	BON1	5.335	1.250	0.807	0.876	0.639	0.876
	BON2	5.340	1.241	0.770			
	BON3	5.330	1.268	0.798			
	BON4	5.333	1.281	0.821			
Customer-perceived value (CPV)	CPV1	4.762	1.569	0.773	0.865	0.616	0.865
	CPV2	4.744	1.579	0.785			
	CPV3	4.766	1.573	0.773			
	CPV4	4.746	1.580	0.807			
Willingness to use (WTU)	WTU1	4.995	1.308	0.775	0.858	0.602	0.858
	WTU2	4.997	1.256	0.765			
	WTU3	4.997	1.273	0.789			
	WTU4	4.977	1.296	0.774			

Note: model fit indices: χ^2^/df = 1.533, CMIN = 245.266, df = 160, IFI = 0.988, CFI = 0.987, TLI = 0.985, and RMSEA = 0.030.

**Table 6:** Mediation analysis: indirect effects and confidence intervals for WTU variables.

	Indirect Effect	Boot SE ^a^	BLLCI ^b^	BULCI ^c^
AUT to WTU	0.182	0.038 ***	0.117	0.269
GAM to WTU	0.199	0.037 ***	0.133	0.280
BON to WTU	0.119	0.036 **	0.053	0.196

Note: ^a^ boot SE: bootstrap standard error, ^b^ BLLCI: bootstrap lower limit confidence interval, and ^c^ BULCI: bootstrap upper limit confidence interval. *p* < 0.05, ** *p* < 0.01, and *** *p* < 0.001.

## Data Availability

The raw data supporting the conclusions of this article will be made available by the authors on request.
